# Definition and diagnosis of postsurgical hypoparathyroidism after thyroid surgery: meta-analysis

**DOI:** 10.1093/bjsopen/zrac102

**Published:** 2022-09-02

**Authors:** Kathrin Nagel, Anne Hendricks, Christina Lenschow, Michael Meir, Stefanie Hahner, Martin Fassnacht, Armin Wiegering, Christoph-Thomas Germer, Nicolas Schlegel

**Affiliations:** Department of General, Visceral, Transplant, Vascular and Pediatric Surgery, University Hospital Würzburg, Würzburg, Germany; Department of General, Visceral, Transplant, Vascular and Pediatric Surgery, University Hospital Würzburg, Würzburg, Germany; Department of General, Visceral, Transplant, Vascular and Pediatric Surgery, University Hospital Würzburg, Würzburg, Germany; Department of General, Visceral, Transplant, Vascular and Pediatric Surgery, University Hospital Würzburg, Würzburg, Germany; Department of Internal Medicine, Division of Endocrinology and Diabetes, University Hospital, University of Würzburg, Würzburg, Germany; Department of Internal Medicine, Division of Endocrinology and Diabetes, University Hospital, University of Würzburg, Würzburg, Germany; Department of General, Visceral, Transplant, Vascular and Pediatric Surgery, University Hospital Würzburg, Würzburg, Germany; Department of General, Visceral, Transplant, Vascular and Pediatric Surgery, University Hospital Würzburg, Würzburg, Germany; Department of General, Visceral, Transplant, Vascular and Pediatric Surgery, University Hospital Würzburg, Würzburg, Germany

## Abstract

**Background:**

Postsurgical hypoparathyroidism (PH) is the most frequent complication after thyroid surgery. The aim of this systematic review and meta-analysis is to summarize a unifying definition of PH and to elucidate the best possible approach for early detection of PH.

**Methods:**

A systematic review of the literature according to the PICO framework using Embase, PUBMED and the Cochrane library was carried out on 1 December 2021 followed by analysis for risk of bias, data extraction and meta-analysis. All studies addressing the definition of postoperative hypoparathyroidism and/or diagnostic approaches for early detection and diagnosis were included. Case reports, commentaries, non-English articles, book chapters and pilot studies and reviews were excluded.

**Results:**

From 13 704 articles, 188 articles were eligible for inclusion and further analysis. These articles provided heterogeneous definitions of PH. Meta-analysis revealed that postoperative measurements of parathormone (PTH) levels have a higher sensitivity and specificity than intraoperative PTH measurements to predict PH after thyroid surgery. None of the timeframes analysed after surgery within the first postoperative day (POD1) was superior to predict the onset of PH. PTH levels of less than 15 pg/ml and less than 10 pg/ml are both reliable threshold levels to predict the postoperative onset of PH. A relative reduction of mean(s.d.) PTH levels from pre- to postoperative values of 73 (standard deviation 11) per cent may also be predictive for the development of PH. The estimation of calcium levels on POD1 are recommended.

**Conclusion:**

PH is best defined as an undetectable or inappropriately low postoperative PTH level in the context of hypocalcaemia with or without hypocalcaemic symptoms. PTH levels should be measured after surgery within 24 h. Both threshold levels below 10 and 15 pg/ml or relative loss of PTH before/after thyroid surgery are reliable to predict the onset of PH.

## Teaser

Actual and comprehensive overview of the definition of postsurgical hypoparathyroidism and meta-analysis on when and how to detect postsurgical hypoparathyroidism. Postsurgical measurements of PTH levels are more sensitive and more specific than intraoperative PTH measurements to predict postsurgical hypoparathyroidism. The end of the operation until 24 h after surgery are the best time points to measure PTH levels to predict postsurgical hypoparathyroidism. PTH levels below 15 and 10 pg/ml have a high sensitivity and specificity to predict the development of postsurgical hypoparathyroidism reliably.

## Introduction

With a rate of 14–60 per cent, postsurgical hypoparathyroidism (PH) is the most frequent complication after thyroid surgery^1–3^. Although most patients seen with PH only have transient problems, there is still a significant number of patients (up to 33 per cent) suffering from persisting hypoparathyroidism (reduced parathyroid hormone (PTH) and calcium levels persisting more than 6 months after thyroid surgery)^2,3^. In a narrative review it has been proposed previously that the time dimension should be incorporated when describing PH. Based on this, PH includes the syndromes of postoperative parathyroid failure, protracted hypoparathyroidism and permanent hypoparathyroidism^4^.

The symptoms of hypoparathyroidism are extremely variable and range from no symptoms to mild numbness and tingling, muscle cramps, tetany, seizures and life-threatening laryngospasm and cardiac arrhythmia.

In most cases, the onset of PH is within the first 48 h after thyroid surgery^5–8^; however, it has also been reported that the first symptoms of hypocalcaemia begin much later, up to 64 h after surgery^9–11^. This has also been described as postoperative parathyroid failure^4^. In view of this, ongoing efforts to discharge patients 24–48 h after thyroid surgery can lead to significant danger for patients, as they will not receive adequate and timely therapeutic intervention if they develop symptoms later. This concern requires a standardized follow-up of patients after thyroid surgery and the earliest possible detection of PH. It would also be desirable to have reliable markers to predict the potential onset of PH to start a (preventive) therapeutic intervention before patients get symptomatic. This is important because a standard ‘blind’ substitution of calcium and vitamin D with the aim of preventing the onset of symptoms has been reported to be one of the main risk factors of developing PH, as the physiological trigger for PTH secretion is blocked^12^.

All these aspects have been addressed by numerous studies so far. Accordingly, there is a large body of literature focusing on how to diagnose PH at the earliest possible time point to decide whether therapeutic intervention is required; however, because even the definition of PH is heterogeneous and there are many different studies addressing this, there remains uncertainty about the appropriate approach for early recognition of PH.

A systematic analysis of the literature on postsurgical hypoparathyroidism was therefore conducted focusing on the following issues: the most common definitions of PH were systematically analysed and discussed. Next, the question of whether there is an ideal time point for early detection of PH or postoperative parathyroid failure respectively, was assessed. Finally, the best possible predictive approach for early recognition of PH was determined.

## Materials and methods

### Search strategy

A systematic review of the literature was conducted according to the PRISMA guidelines^13^. The review protocol was registered at Prospero (https://www.crd.york.ac.uk/); PROSPERO 2022 CRD42022303713.

To get a comprehensive overview of the existing body of literature, a systematic literature search of PubMed via MEDLINE, Embase and the Cochrane library electronic databases was performed on 1 December 2021. The timeframe of the literature search was from the overall start of documentation in the databases until 30 November 2021. A systemic analysis of the literature was conducted according to the PICO framework^14^. According to this, ‘patients after thyroid surgery’ were defined as the population and ‘postsurgical hypoparathyroidism’ as the phenomenon of interest, and ‘diagnostics’ as the context. All search terms were assigned into these three subgroups (*Table S1*). The words ‘AND’ and ‘OR’ were used as Boolean operators. To increase the sensitivity of the literature search, the following medical subject heading terms were included: ‘hypoparathyroidism’, ‘hypocalcemia’, ‘postoperative complications’, ‘postoperative period’, ‘thyroidectomy’, ‘parathyroid hormone’, ‘hemithyroidectomy’ and ‘subtotal thyroidectomy’. Again ‘AND’ and ‘OR’ were used as Boolean operators.

All studies with abstracts in the English language were included. Duplicates were removed by the literature organization program in addition to manual control. Two independent reviewers (K.N. and N.S.) performed the screening of titles and abstracts of all studies. Potentially relevant articles were reviewed in full to determine eligibility for inclusion. Data for meta-analysis were extracted by one author (K.N.) and double checked by the other authors (A.H. and N.S.). In case of missing/incomplete data, the study investigator was contacted for additional details. Any disagreement was discussed and solved by consensus among the authors.

### Study selection criteria

All studies addressing the definition of PH and/or diagnostic approaches for early detection and diagnosis were included. Both, prospective and retrospective studies were included. Case reports, commentaries, non-English articles, book chapters and pilot studies and reviews were excluded. Conference proceedings and unpublished studies were included if they provided sufficient information. If two studies examined the same study population, the more recent study was included.

For meta-analyses, all studies were manually screened and compared regarding whether they displayed a comparable study design and equal outcome parameters. This is outlined in detail in the results for the respective topic addressed.

### Data management, risk of bias assessment and statistical analysis

The literature organization was performed with Endnote20™ (Clarivate Analytics, Munich, Germany). Charts and tables were created with Microsoft^®^ Word and Microsoft^®^ PowerPoint (Microsoft, Redmond, Washington, USA), and RevMan5 (Cochrane Community). The studies included for meta-analysis were assessed for the risk of bias using the ROBINS-I tool^15^ (*Table S2*).

To compare PTH levels and calcium levels and to provide a comprehensive overview, units were adapted for PTH in pg/ml and for calcium in mmol/l. The calculation was performed using the calculator provided in unitslab.com.

Statistical analysis was performed with SPSS^®^ version 26 (IBM, Armonk, New York, USA), RevMan5 and OpenMeta (Analyst)^16^. As a measure of effects, bivariate analysis for sensitivity and specificity with the corresponding 95 per cent confidence interval (c.i.) was calculated.

## Results

**Fig. 1:**
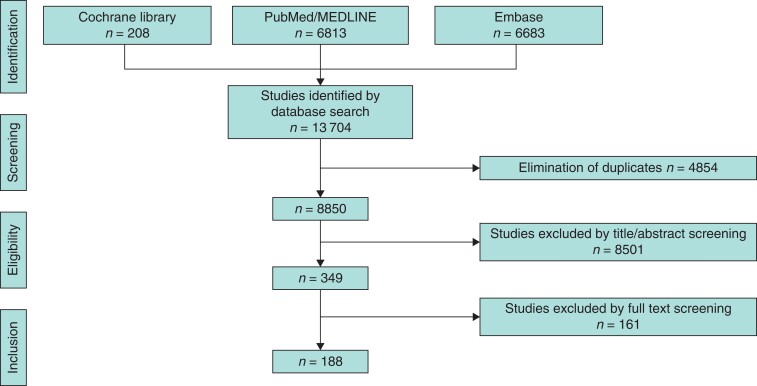
PRISMA flowchart for the literature search

The database search identified 13 704 articles. After removing duplications, 8850 articles were screened for eligibility and inclusion in the systematic review (*Fig. 1*). After exclusion of studies by title/abstract and full text screening, 188 articles were eligible for inclusion in this review. After this, all articles were analysed in depth according to the main foci of this review. This led to a variable number of studies included for the different subheadings, stated below. Analyses for risk of bias in the studies included in the meta-analyses are shown in *Table S2*.

### Definition of postsurgical hypoparathyroidism

According to the focus ‘definition of PH’, 188 articles identified in the database were assessed for eligibility. From these, 31 studies were excluded because hypoparathyroidism was not defined, or no clear definition was stated so that 157 articles including 29 346 patients were subject to further analysis.

As shown in *Table 1*, these studies could be assigned into four subgroups. The definitions of PH included reduced PTH levels only, hypocalcaemia only, reduced PTH or hypocalcaemia or a combination of both. In most cases, these groups could be subdivided into studies that included the presence or absence of symptoms to define the presence of PH. Taken together, this confirmed that there is a large heterogeneity of definitions of PH in the different studies. Therefore, direct systematic comparisons of the parameters discussed below are nearly impossible.

**Table 1:** Studies identified with specific definitions of postsurgical hypoparathyroidism

Definition criteria of postsurgical hypoparathyroidism	Number of patients included	References
**Reduced PTH levels *n* = 3**		
Biochemical alterations only	461	^17–19^
**Hypocalcaemia AND reduced PTH levels *n* = 2**		
Biochemical alterations only	1172	^20,21^
**Hypocalcaemia OR reduced PTH levels *n* = 20**		
Biochemical alterations only *n* = 8	2563	^22–29^
Symptoms independent from biochemical hypocalcaemia *n* = 2	486	^30,31^
Biochemical alterations and/or symptoms *n* = 10	2920	^32–41^
**Hypocalcaemia only *n* = 132**		
Biochemical alterations only *n* = 42	6475	^6,7,42–81^
Symptoms independent from hypocalcaemia *n* = 20	4258	^82–101^
Hypocalcaemia and/or symptoms *n* = 63	9936	^10,102–163^
Hypocalcaemia and symptoms *n* = 7	1075	^164–170^

The main criteria and the sub-definitions within these categories are listed in the first column; *n* indicates the number of studies found. Reduced PTH and calcium indicates levels below the level of normal; whether calcium levels referred to adjusted, ionized or total calcium was reported in the studies could not be identified in all studies so that they were taken together as ‘hypocalcaemia’. PTH, parathyroid hormone.

In addition, the lower limits of serum calcium levels seem slightly heterogeneous depending on the local laboratories^171^. Forty studies including 7718 patients defined hypocalcaemia according to the lower limit of normal of the local laboratories in which their measurements were carried out. This corresponds to the recommendation of the American Association of Clinical Endocrinology^172^. The majority of 52 studies including 11 504 patients, defined hypocalcaemia as a calcium level below the lower limit of 2.0 mmol/l (less than 8.0 mg/dl), which is in accordance with other recommendations^8,173^. It must be considered whether uncorrected serum calcium levels, albumin-corrected serum calcium levels or ionized calcium levels are the basis for the different studies to define hypocalcaemia. In addition, there is evidence that patients with calcium values less than 2.0 mmol/l may develop symptoms of hypocalcaemia^102,174^. In view of all these aspects, both approaches to define hypocalcaemia seem justified although they do not necessarily correspond to clinical symptoms.

On the other hand, the reduction of PTH levels is a good predictor for symptomatic hypoparathyroidism. It has been shown that it is more sensitive at detecting patients at risk and detecting them earlier because loss of PTH precedes biochemical hypocalcaemia^82,103,104^. Therefore, based on the literature search, the diagnosis should be predominately oriented on the early biochemical changes of PTH levels after surgery.

When addressing the clinical picture caused by hypoparathyroidism, a large inter-individual variety was found ranging from ‘no symptoms’ in patients with clear biochemical evidence for the presence of PH to a group of patients with ‘potentially life-threatening symptoms’ because of laryngospasm, muscle cramps or cardiac arrhythmia with biochemical changes that were mild at the time when symptoms started^104,175^. Therefore, it was concluded that the presence of clinical symptoms seemed to not be applicable to define PH. Furthermore, changes in calcium and PTH levels and their interdependence need to be considered to define PH.

Taking all these considerations into account, the following definition is suggested: PH can be defined as an undetectable or inappropriately low postoperative PTH level in the context of hypocalcaemia with or without hypocalcaemic symptoms. In this definition, the term ‘inappropriately low’ is thought to reflect the strong interdependence between PTH and calcium levels as even PTH in lower-normal ranges may be inappropriate to maintain normal calcium levels. Therefore, hypoparathyroidism may be present even when PTH levels seem to be in the normal range. As the development of symptoms is subjective, it remains unclear and not clearly quantifiable how low PTH levels can be to be considered as inappropriately low. It can be speculated whether the ratio between PTH and calcium levels is correlated with symptoms, and a prospective study specifically designed to address this question would be required.

### Suitable time point to predict postsurgical hypoparathyroidism based on PTH levels

The significant correlation of reduced PTH levels with the manifestation of PH following thyroid surgery is well established. Many studies have aimed to determine a suitable time point for intraoperative or postoperative PTH measurements to predict the development of PH as early and precisely as possible.

The first aim was to determine whether intraoperative or postoperative PTH measurements are superior for early and specific prediction of developing PH. Intraoperative PTH measurements were usually carried out between 10 and 20 min after thyroidectomy or at the time point when surgery ended with skin closure.

**Fig. 2:**
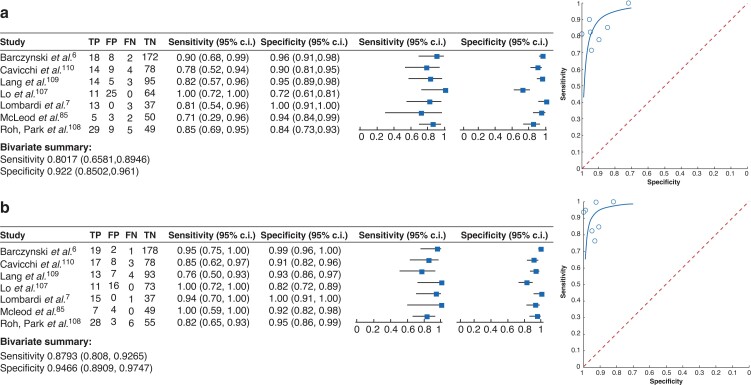
Forest plot (left) and summary receiver operating characteristic curves (right) Studies analysing the value of **a** intraoperative parathyroid hormone measurements and **b** postoperative parathyroid hormone measurements to identify postsurgical hypoparathyroidism. The outcome parameter is hypocalcaemia in the presence or absence of symptoms as indicated by the study protocol of the studies included. Bivariate analysis summarized the sensitivity and specificity for each condition. TP, true positive; FP, false positive; FN, false negative; TN, true negative.

The direct comparison between intra- and postoperative PTH measurements was carried out in a total of 13 studies. Eight articles including 652 patients supported the view that postoperative PTH measurements are more sensitive and more specific compared with intraoperative PTH measurements in predicting PH^7,22,83–85,105,106,176^ (*Table S3*). Three articles including 392 patients did not show a significant difference between intra- and postoperative PTH measurements^6,107,108^ whereas two studies with 223 patients claimed that the intraoperative measurement of PTH is advantageous for early detection of PH^109,110^. Seven of these articles could be summarized in a meta-analysis (*Fig. 2*). The meta-analysis demonstrated a sensitivity of 80 per cent (95 per cent c.i. 0.66 to 0.90) and a specificity of 92 per cent (95 per cent c.i. 0.85 to 0.96) for intraoperative measurements of PTH values to be predictive for PH. However, with a sensitivity of 87 per cent (95 per cent c.i. 0.81 to 0.93) and a specificity of 95 per cent (95 per cent c.i. 0.89 to 0.98), the postoperative measurement of PTH levels seems to be superior compared with the intraoperative measurement of PTH levels to predict PH. Despite an overlap of confidence intervals, this seems to support the view of most studies that postsurgical measurements of PTH levels can be recommended for a reliable detection of PH rather than intraoperative measurements.

**Fig. 3:**
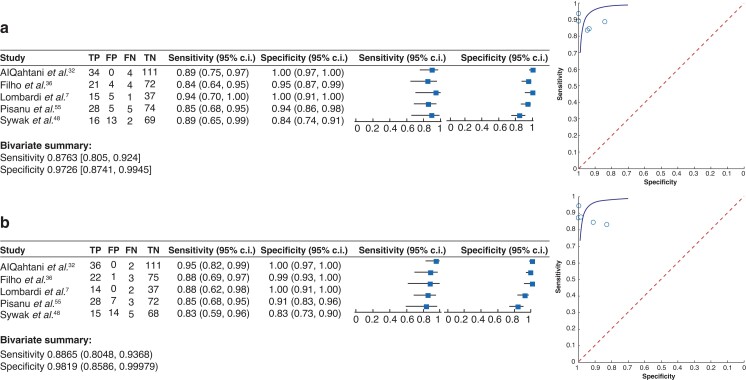
Forest plot (left) and summary receiver operating characteristic curves (right) Studies analysing **a** predictive value of early postoperative (1–6 h) and **b** later postoperative (24 h/POD1) parathyroid hormone measurements to identify postsurgical hypoparathyroidism. The definition of hypoparathyroidism varied between studies. Only studies were included in the meta-analysis that investigated both time points in the same cohort of patients. Bivariate analysis summarized the sensitivity and specificity for each condition. TP, true positive; FP, false positive; FN, false negative; TN, true negative.

Next, the focus was on articles that performed postoperative PTH measurements with the aim of determining the best time point after thyroid surgery to detect PH. Overall, 21 articles were found to address the most suitable time point for PTH measurements ranging from 1 h and 6 h until 24 h after surgery or within the first postoperative day (POD1) (*Table 2*). All articles identified a time point of PTH measurement that was reported to be advantageous for early detection of PH, however none of the studies reported statistical significance when different time points were compared. In addition, there was extreme heterogeneity concerning study design, outcomes reported, and time points investigated. Therefore, only five studies were suitable to compare the sensitivity and specificity of two timeframes of postoperative PTH measurements, as they allowed direct comparison of the reported data (*Fig. 3*). It was decided to compare the timeframe within the first 6 and 24 h or within POD1 for PTH measurements after thyroid surgery as these timeframes were assessed most often in the literature and can result in clinical consequences. The early timeframe for measurements of PTH levels within 1–6 h after thyroid surgery resulted in an overall sensitivity of 88 per cent (95 per cent c.i. 0.81 to 0.92) and a specificity of 97 per cent (95 per cent c.i. 0.87 to 1.00) in predicting PH. The analysis of the later timeframe included data on post-surgical PTH measurements after 24 h or within POD1, which provided an overall sensitivity of 89 per cent (95 per cent c.i. 0.81 to 0.94) and a specificity of 98 per cent (95 per cent c.i. 0.86 to 1.00) in predicting PH. In summary, both timeframes resulted in almost equally high sensitivity and specificity values with small 95 per cent c.i. ranges in detecting PH. The conclusion therefore is that there is no distinct time after surgery that can be recommended for PTH measurements.

**Table 2:** Overview of studies reporting the superiority of a defined time point for postoperative parathyroid hormone measurements

Reference	Year of publication	Study design	Number of patients included	Time point(s) of PTH measurements	Statistical significance
**Lam & Kerr^42^**	2003	Prospective	40	1, 6 h	NA
**Payne *et al*.^a141,177^**	2003	Prospective	54	12 h	NA
**Lombardi *et al*.^7^**	2004	Prospective	53	SC, 2, 4, 6, 24, 48 h	NA
**Vescan *et al*.^112^**	2005	Prospective	199	1 h, POD1	NA
**Payne *et al*.^a^ ^125^**	2005	Prospective	70	6h	NA
**Roh & Park^108^**	2006	Prospective	92	SC,1 h, POD1	NA
**Sywak *et al*.^48^**	2007	Prospective	100	4, 23 h	No difference
**Al-Dhahri *et al*.^132^**	2010	Retrospective	79	6, 12, 20, 32, 44h	No difference
**J. H. Kim, Chung, & Son^104^**	2011	Retrospective	112	1 h, POD1	NA
**Kim *et al*.^83^**	2013	Prospective	108	SC, 6, 12, 24, 48, 72 h	NA
**Pisanu *et al*.^55^**	2013	Prospective	112	6, 24, 48 h	NA
**Al-Dhahri *et al*.^164^**	2014	Prospective	168	1, 6 h	No difference
**AlQahtani *et al*.^32^**	2014	NA	149	1, 6, 24 h	No difference
**Carr *et al*.^90^**	2014	Retrospective	77	4 h, POD1	No difference
**Schlottmann *et al*.^111^**	2015	Prospective	106	1, 3, 6 h	No difference
**Sieniawski *et al*.^20^**	2016	Prospective	142	1, 6 h	No difference
**Yetkin *et al*.^33^**	2016	Prospective	202 (SG)	1, 24 h	No difference
			+72 (CG)		
**White *et al*.^34^**	2016	Prospective	196	1 h, POD1	No difference
**Arer *et al*.^96^**	2017	Prospective	106	6, 12, 24 h	No difference
**Filho *et al*.^36^**	2018	Prospective	101	1–4 h, POD1	No difference

Most of the studies described one superior time point for PTH measurements but none of them reported that the differences were statistically significant. PTH, parathyroid hormone; SC, PTH measurement skin closure; POD1, measurement postoperative day 1; NA, no data on statistically significant difference available: SG, study group; CG, control group as defined in the publication.

a Studies can only be interpreted in combination, cited together.

It is reasonable that earlier measurements of PTH levels in the postoperative course will enable the early recognition of a potential problem and may lead to an indication for earlier therapeutic administration of calcium and vitamin D medication before patients develop symptoms. This is supported by 10 additional articles that could not be included in the meta-analysis showing the predictive value of PTH measurements 1 h after surgery is comparable to later time points^20,32–34,42,104,108,111,112,164^. This supports the main conclusion of the meta-analysis. The decision of which standard is the most applicable, however, will depend on the specific local facilities as PTH measurements may not be available outside core working hours.

### Threshold levels for PTH to predict postsurgical hypoparathyroidism

The main problem in defining threshold levels for PTH is that there are different assays that result in different normal ranges of PTH levels^178^. This is supported by the observation that 81 studies defined 40 different PTH levels as the most reliable threshold to detect PH (*Table S4*). Therefore, to avoid assay-related confusion in preoperative PTH and calcium measurements, it is suggested that both PTH and calcium levels should be estimated by the same laboratories/institutions where postsurgical measurements will take place.

**Fig. 4:**
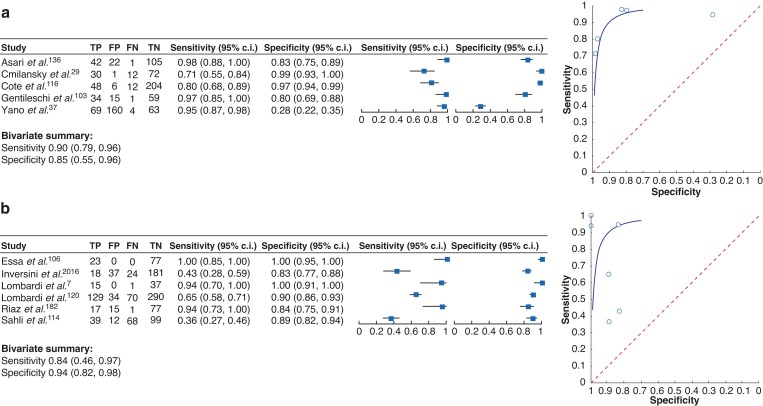
Forest plot (left) and summary receiver operating characteristic curves (right) Studies of parathyroid hormone threshold levels of **a** less than 15 pg/ml and **b** less than 10 pg/ml to identify postsurgical hypoparathyroidism (development of hypocalcaemia). Bivariate analysis summarized the sensitivity and specificity for each condition. TP, true positive; FP, false positive; FN, false negative; TN, true negative.

**Fig. 5:**
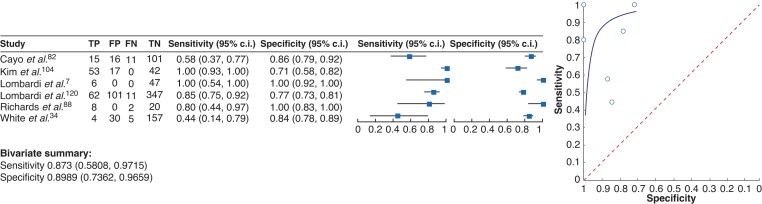
Forest plot (left) and summary receiver operating characteristic curves (right) Studies of parathyroid hormone threshold levels of less than10 pg/ml to identify postsurgical hypoparathyroidism (development of symptoms) are shown. Bivariate analysis summarized the sensitivity and specificity for each condition. TP, true positive; FP, false positive; FN, false negative; TN, true negative.

In addition, 20 articles were identified that analysed the diagnostic value of their lower limits of normal and included them in the more detailed analysis of the most commonly identified thresholds. In summary, 55 articles were eligible for further analysis. Seven articles that tested the predictive value of PTH levels below 20 pg/ml^23,43,44,86,113–115^, 19 articles on threshold levels below 15 pg/ml^24,45–47,87,108,116–119^ and 29 articles^6,34,48,49,82,88,89,104,106,113,114,120–124,179,180^ that tested the predictive value of PTH threshold levels below 10 pg/ml were identified. Out of these articles, the studies that aimed to identify PTH threshold levels as isolated parameters to predict PH were selected (*Table 3*). For each of the threshold levels of 10 pg/ml and 15 pg/ml, five studies were identified that analysed this aspect with a comparable study setting for further analyses (*Fig. 4*). The other studies could not be included because the study design was different, or data could not be extracted. It was not possible to summarize studies for the threshold level of 20 pg/ml due to their heterogeneity. When summarizing the results from the studies for 15 pg/ml (*Fig. 4a*) and 10 pg/ml (*Fig. 4b*), threshold levels of less than 15 pg/ml were found to have a sensitivity of 90 per cent (95 per cent c.i. 0.79 to 0.96) and a specificity of 85 per cent (95 per cent c.i. 0.55 to 0.96) in predicting PH. The threshold level of PTH values less than 10 pg/ml had a sensitivity of 84 per cent (95 per cent c.i. 0.46 to 0.97) and specificity of 94 per cent (95 per cent c.i. 0.82 to 0.98) in predicting PH. In an additional meta-analysis with studies that used threshold levels of 10 pg/ml to predict symptoms of hypoparathyroidism, a sensitivity of 87 per cent (95 per cent c.i. 0.58 to 0.97) and a specificity of 90 per cent (95 per cent c.i. 0.74 to 0.97) were found (*Fig. 5*). This led to the conclusion that both threshold levels are suitable to reliably predict the onset of PH. Taken together, using a threshold level that is oriented at the assay-specific lower limit of normal for PTH will lead to a high specificity and sensitivity for early detection of PH.

**Table 3:** Overview of studies focusing on threshold levels of 10 pg/ml, 15 pg/ml and 20 pg/ml, including study design, number of patients, time point of parathyroid hormone measurement following thyroid surgery and endpoint to identify postsurgical hypoparathyroidism

Threshold levels of PTH	Reference	Year of publication	Study design	Number of patients included	Lower limit of normal PTH (pg/ml)	Time point of PTH measurement	Endpoint
**Threshold levels of PTH less than 10 pg/ml**	Richards *et al*.^88^	2003	Prospective	30	12 pg/ml	SC	Symptoms
	Lombardi *et al*.^7^	2004	Prospective	53	10 pg/ml	4 h/6 h	Ca^2+^<2.0 mmol/l Symptoms
	Quiros *et al*.^181^	2005	Prospective	72	10 pg/ml	SC	Ca^2+^<2.1 mmol/l Symptoms
	Lombardi *et al*.^120^	2006	Prospective	523	10 pg/ml	4 h	Ca^2+^<2.0 mmol/l Symptoms
	Barczyński *et al*.^6^	2007	Prospective	200	10 pg/ml	4 h	Ca^2+^<2.0 mmol/l
	Gentileschi *et al*.^103^	2008	Prospective	119	15 pg/ml	1 h	Symptoms
	Youngwirth *et al*.^166^	2010	Retrospective	371	10 pg/ml	4 h/POD1	Ca^2+^<2.1 mmol/l Symptoms
	Wiseman *et al*.^46^	2010	Retrospective	421	11 pg/ml	1 h	Ca^2+^<2.2 mmol/l
							Ca^2+^<1.9 mmol/l
	Kim *et al*.^104^	2011	Retrospective	112	11 pg/ml	1 h	Symptoms
	Cayo *et al*.^82^	2012	Prospective	147	Not reported	POD1	Symptoms
	Riaz *et al*.^182^	2014	Not reported	110	Not reported	1 h	NA
	White *et al*.^34^	2016	Prospective	196	15 pg/ml	1 h	Ca^2+^<2.0 mmol/l Symptoms
	Inversini *et al*.^124^	2016	Retrospective	260	Not reported	3–6 h	Ca^2+^<2.0 mmol/l Symptoms
	Al Khadem *et al*.^122^	2018	Retrospective	119	10 pg/ml	PACU	Ca^2+^<2 mmol/l Symptoms
	Sahli *et al*.^114^	2018	Prospective	218	10 pg/ml	1 h	iCa^2+^<1.13 mmol/l Symptoms
	Essa *et al*.^106^	2021	Prospective	100	15 pg/ml	10 min after TT	Ca^2+^<2.1 mmol/l Symptoms
	Abdullah *et al*.^49^	2021	Retrospective	57	10 pg/ml	3 h	Ca^2+^<2.1 mmol/l
**Threshold levels of PTH less than 15 pg/ml**	Warren *et al*.^117^	2002	Retrospective	53	Not reported	IntraOp	iCa^2+^<1.0 mmol/l Symptoms
	Chia *et al*.^118^	2006	Prospective	103	Not reported	8 h	Ca^2+^<1.9 mmol/l Symptoms
	Ghaheri *et al*.^47^	2006	Retrospective	80	Not reported	PACU	iCa^2+^<1.0 mmol/l
	Chindavijak *et al*.^58^	2007	Prospective	30	15 pg/ml	IntraOp	Ca^2+^<2.1 mmol/l Symptoms
	Lewandowicz *et al*.^17^	2007	Prospective	54	15 pg/ml	SC	Ca^2+^< 2.1 mmol/l PTH
	Cote *et al*.^116^	2008	Retrospective	270	Not reported	1 h	Ca^2+^<2.0 mmol/l Symptoms
	Gentileschi *et al*.^103^	2008	Prospective	119	15 pg/ml	1 h	Ca^2+^<2.0 mmol/l Symptoms
	Asari *et al*.^136^	2008	Prospective	170	15 pg/ml	POD1	cCa^2+^<2.0 mmol/l Symptoms
	Huang *et al*.^24^	2012	Prospective	197	15 pg/ml	IntraOp	Ca^2+^<2.0 mmol/l
	Yano *et al*.^37^	2012	Retrospective	296	15 pg/ml	POD1	cCa^2+^<2.0 mmol/l Symptoms
	Islam *et al*.^45^	2013	Prospective	65	12 pg/ml	IntraOp	Ca^2+^<2.0 mmol/l Symptoms
	Cmilansky *et al*.^29^	2014	Prospective	115	15 pg/ml	POD1	Ca^2+^<2.0 mmol/l Symptoms
	Yetkin *et al*.^33^	2016	Prospective	274	15 pg/ml	1 h	Ca^2+^<2.0 mmol/l Symptoms
**Threshold levels of PTH less than 20 pg/ml**	Sabour *et al*.^44^	2009	Retrospective	448	15 pg/ml	PACU	cCa^2+^<2.0 mmol/l cCa^2+^<1.9 mmol/l
	Proczko-Markuszewska *et al*.^43^	2010	Prospective	100	10 pg/ml	1 h	Ca^2+^<2.0 mmol/l Symptoms
	Houlton *et al*.^23^	2011	Retrospective	180	15 pg/ml	PACU	Ca^2+^<2.0 mmol/l
	Noureldine *et al*.^113^	2014	Retrospective	304	10 pg/ml	6–8 h	Symptoms Ca^2+^<2.0 mmol/l
	Lee *et al*.^86^	2015	Prospective	817	Not reported	1 h	Symptoms
	Sahli *et al*.^114^	2018	Prospective	218	10 pg/ml	1 h	iCa^2+^<1.1 mmol/l Symptoms
	Bashir *et al*.^115^	2021	Prospective	175 (phase 1)	14.9 pg/ml	Immediately after surgery	Ca^2+^<2.0 mmol/l Symptoms

If required calcium and PTH values were adapted to pg/ml or mmol/l respectively. PTH, parathyroid hormone; POD1, postoperative day 1; IntraOp, measurement during surgery; cCa^2+^, corrected calcium; iCa^2+^, ionized calcium; PACU, post-anaesthesia care unit; NA, not available; SC, PTH measurement skin closure; TT, total thyroidectomy.

### Relative reduction of pre- and postoperative PTH levels to predict postsurgical hypoparathyroidism

In view of the difficulties comparing different PTH assays it was proposed that a ratio between preoperative and postoperative PTH value may be suitable to reliably predict the manifestation of PH. In the literature, 51 articles were identified that focused on the relative reduction of PTH levels when pre- and postoperative measurements were compared. Looking at these articles systematically, 29 different ratios between preoperative and postoperative PTH values were found that were reported to predict PH. These ranged between a relative reduction of PTH preoperative/postoperative values of 19.4 per cent^22^ and 88.0 per cent^183^. Two studies sought to optimize the predictive value by forming risk groups in addition to the relative reduction of PTH levels after surgery, which resulted in the highest sensitivity and specificity to predict patients with PH^122,164^. This approach, however, seems to not be applicable in daily clinical practice. One of the main problems in determining a ratio between pre- and post-surgical PTH levels is that the time points of PTH measurements vary considerably in each study. As PTH levels show rapid changes under physiological conditions it can be impossible to exactly standardize time points of PTH measurements in daily routine. This is exemplified by a study that assessed PTH levels in 74 patients undergoing thyroidectomy before induction of anaesthesia, after induction of anaesthesia, 20 min after thyroidectomy and in the postoperative course^184^. This showed that during induction of anaesthesia, there is a relevant but unpredictable dynamic of PTH that changed to 149 (standard deviation 93) per cent of baseline levels (range 42–49.4 per cent) and normalized during surgery.

A meta-analysis by Noordzij *et al.* analysed nine studies to assess in more detail whether the relative loss of PTH before, during and after surgery can predict PH^5^. In 85 patients, a loss of more than 65 per cent of PTH levels compared before and 6 h after surgery had a sensitivity of 96.4 per cent and a specificity of 91.2 per cent to adequately predict PH.

**Fig. 6:**
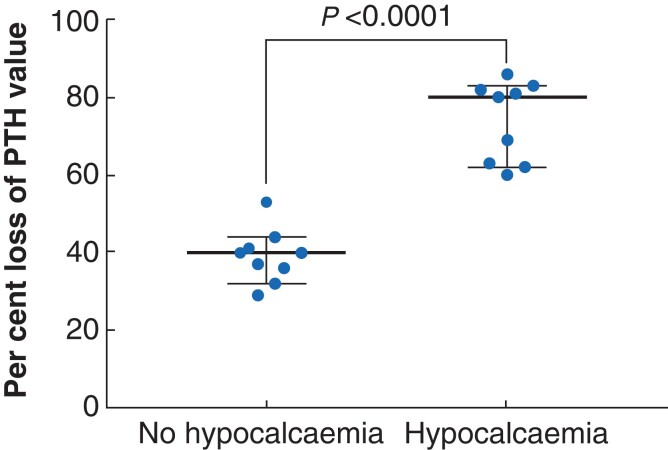
Median of mean and 95 per cent confidence intervals from values of relative reduction of parathyroid hormone levels from eight comparable studies These were extracted to assess whether these values can be used to predict hypocalcaemia in patients after thyroid surgery. All studies together represent a cohort of 1358 patients. Unpaired non-parametric Kruskal–Wallis test was used to test for significant differences. PTH, parathyroid hormone.

In further analyses, eight original articles (*Table 4*) with comparable features in terms of pre- and postsurgical setting for PTH measurements were identified. Studies in which PTH measurements had been carried out before induction of anaesthesia were compared. When taking them together, a mean reduction of PTH levels of 73 ± 11 per cent was observed in the patient cohort that developed hypocalcaemia, whereas the group of patients with a mean reduction of PTH levels of 39.5 ± 7.3 per cent had no hypocalcaemia in the following course (*P* < 0.0001; *Fig. 6*).

**Table 4:** Overview of the studies that tested the predictive value when the relative loss of parathyroid hormone levels between pre- and postoperative levels were compared

Reference	Year of publication	Number of patients included	Mean postoperative reduction of PTH levels	
			Patients without hypocalcaemia (%)	Patients with hypocalcaemia (%)
**Roh/Park *et al*.^108^**	2006	92	37	81
**Barczinsky *et al*.^6^**	2007	200	32	69
**Toniato *et al*.^69^**	2008	160	40	63
**Mehrvarz *et al*.^127^**	2014	99	41	60
**Puzziello *et al*.^56^**	2015	75	44	62
**Seo *et al*.^59^**	2015	349	49	80
**Sieniawski *et al*.^20^**	2016	142	36	82
**Suwannasarn *et al*.^54^**	2017	65	29	83
**Mo *et al*.^170^**	2020	176	53	86
**Mean(s.d.)**		1358	39.5(7.3)	73.0(11)

PTH, parathyroid hormone.

Based on this and on the results of the meta-analysis described above, it can be concluded that a relative reduction of PTH of more than 70 per cent after surgery can be predictive for the development of PH. On the other hand, this should be considered with caution as, in addition to the measurement uncertainty, other physiological factors, including vitamin D status may affect the relative loss of PTH levels after surgery. The relationship between preoperative vitamin D status and the development of PH is controversial as there are a number of manuscripts supporting this^185–190^, and some that do not show a significant relationship^35,191–199^. Due to this, all studies independent of the vitamin D status were included in this meta-analysis.

### Role of postsurgical calcium measurements

It goes without saying, that PTH measurements do not replace the need to control postsurgical calcium levels. Therefore, it is broadly accepted and recommended that calcium measurements should be carried out after surgery at least on POD1, whereas some favour including measurements on POD2^9,200^. In cases, in which PTH levels have been measured at appropriate levels after surgery and calcium levels on POD1 are in the normal range, it may be discussed that the control of calcium levels on POD2 are dispensable. This is supported by three articles confirming that the combination of early PTH measurements and calcium estimation on POD1 is a safe procedure for patients to reliably identify those patients who will not develop PH^123,125,126^.

In summary, based on the present review of the literature, structured surveillance of perioperative parathyroid function in thyroid surgery is recommended, which should include early postsurgical PTH values to decide on (prophylactic) therapeutic intervention that should be completed by estimation of calcium levels at least on POD1. In cases of abnormal results such as low levels of calcium and PTH or hypocalcaemic symptoms, measurements of calcium should be repeated on POD2.

## Discussion

The literature provides a lot of heterogeneous and observational studies focusing on the problem of PH. In the future, a consensus-based uniform definition for PH should be developed to provide the basis for future studies and clinical application. Based on this review and meta-analysis and keeping the mentioned limitations in mind, the key conclusions and suggestions are as follows:

- PH can be defined as an undetectable or inappropriately low postoperative PTH level in the context of hypocalcaemia with or without hypocalcaemic symptoms.
- Postsurgical measurements of PTH levels have a higher sensitivity and specificity than intraoperative PTH measurements in predicting PH.
- The ideal time point of the measurements of postsurgical PTH levels to predict PH is between the end of the operation until 24 h after surgery. There are no significant differences within this timeframe.
- Serum PTH levels as a threshold for detecting PH most often corresponded to the lower levels of normal of laboratories where PTH measurements were carried out. According to the meta-analysis, PTH levels below 15 and 10 pg/ml give a high sensitivity and specificity in predicting the development of PH. Using a threshold level that is oriented at the assay-specific lower limit of normal for PTH for early detection of PH is suggested.
- A PTH decrease from pre- to postoperative sampling of more than 73 ± 11 per cent seems to predict the development of PH, provided that the preoperative measurements are carried out in the same laboratory and before induction of anaesthesia. In addition to the measurement uncertainty and other physiological factors including the vitamin D status, the reliability of the relative reduction of PTH should be used with caution.
- Independent from PTH measurements, the estimation of calcium levels on POD1 should be carried out. Additional calcium measurements may not be required if PTH and calcium values are normal in the early postoperative course and patients do not develop symptoms^201–203^.

## Supplementary Material

zrac102_Supplementary_Data

## Data Availability

The data of this review and meta-analysis can be made available to any researcher. All relevant data are included in the tables and supplemental material. All other material and data can be provided directly on request to the corresponding author.
