# Supplementary material for: Definition and diagnosis of postsurgical hypoparathyroidism after thyroid surgery: meta-analysis
Source: BJS Open. 2022 Sep 2;6(5):zrac102. doi: 10.1093/bjsopen/zrac102 (PMC9437325; doi:10.1093/bjsopen/zrac102)
Supplement: zrac102_Supplementary_Data [file zrac102_supplementary_data.docx]

**Table S1:**

| **Population** | **Phenomenon of Interest** | **Context** |
| --- | --- | --- |
| complication,postoperative  complication,surgical  post operative complication  post operative complications  postoperative complication  postoperative complications  postsurgical complication  surgical complication  thyroidectomy  thyroid surgery  postsurgical  thyroid operation  post thyroidectomy  postoperative period  postoperative phase | hypoparathyroidism  hypoparathyreose  hypoparathyroid  parathyroid hypofunction  parathyroid insufficiency  hypoparathyroid dysfunction  hypocalcemia  hypocalcaemic activity  hypocalcemic activity  secondary hypocalcaemia  secondary hypocalcemia  hypocalcemic symptoms  tetany  muscle tetany  tetanic  muscle cramp  cramp  cramp,muscle  cramps  muscular cramp  muscle spasm  diffuse vascular spasm  involuntary muscle contraction  muscular spasm  myospastic  myospasm  spasm  spasm,muscle  „chvostek sign“  „trousseau sign“ | diagnosis  diagnostic screening  diagnostic sign  diagnostic tool  diagnostics  disease diagnosis  medical diagnosis  physical diagnosis  calcium blood level  blood calcium  calcemia  calcium blood level  calcium,blood  normocalcemic  plasma calcium  plasma free calcium  serum ca  serum calcium  serum free calcium  parathyroid hormone  pth  human parathyroid hormone  parathormone  parathyrin  parathyroid hormones  detection  diagnostic |

The search items for the PICO framework are shown in detail. The subgroups were divided in the subgroups population= patients after thyroid surgery; Phenomenon of Interest = postsurgical hypocalcaemia and postsurgical hypoparathyroidism, Context = Diagnostic criteria; Boolean operators “AND” and “OR” were used to increase the sensitivity.

**Table S2:** Analysis for risk of bias of the studies included in the meta-analysis using the ROBINS-I tool**.**

|  | **Baseline confounding** | **Selection of participants** | **Classification of diagnostic tools** | **Deviation from intended diagnostic** | **Missing data** | **Measurement of outcomes** | **Selection of reported results** | **Overall risk of bias** |
| --- | --- | --- | --- | --- | --- | --- | --- | --- |
| Barczyński, 2007 | moderate | low | low | low | low | low | low | moderate |
| Cavicchi, 2008 | moderate | low | low | low | low | moderate | moderate | moderate |
| Lang,  2012 | moderate | low | low | low | low | moderate | moderate | moderate |
| Lo, 2002 | moderate | low | low | low | low | moderate | low | moderate |
| Lombardi, 2004 | moderate | low | low | low | low | moderate | low | moderate |
| McLeod, 2006 | moderate | low | low | moderate | low | moderate | moderate | moderate |
| Roh, Park, 2006 | moderate | low | low | low | low | moderate | low | moderate |
| AlQuahtani 2014 | moderate | low | low | low | moderate | moderate | moderate | moderate |
| Filho 2018 | moderate | low | low | low | low | moderate | moderate | moderate |
| Lombardi 2004 | moderate | low | low | low | low | moderate | low | moderate |
| Pisanu 2013 | moderate | low | low | low | low | low | moderate | moderate |
| Sywak 2007 | low | low | low | low | low | low | moderate | moderate |
| Asari 2008 | low | low | low | low | moderate | moderate | low | moderate |
| Cmilansky 2014 | moderate | low | low | low | low | moderate | low | moderate |
| Cote 2008 | moderate | low | low | low | moderate | moderate | low | moderate |
| Gentileschi 2008 | moderate | low | low | low | low | low | moderate | moderate |
| Yano 2012 | moderate | low | low | low | low | moderate | serious | serious |
| Inversini 2016 | moderate | low | low | low | low | moderate | moderate | moderate |
| Lombardi 2004 | moderate | low | low | low | low | moderate | low | moderate |
| Lombardi 2006 | moderate | low | low | low | low | low | low | moderate |
| Riaz 2010 | moderate | low | low | low | low | moderate | moderate | moderate |
| Sahli 2018 | moderate | low | low | low | moderate | low | moderate | moderate |
| Cayo 2012 | moderate | low | low | moderate | low | moderate | moderate | moderate |
| Kim 2011 | moderate | low | low | low | low | moderate | Serious | serious |
| Lombardi 2004 | moderate | low | low | low | low | moderate | low | moderate |
| Lombardi 2006 | moderate | low | low | low | low | low | low | moderate |
| Richards 2003 | moderate | Low | low | low | low | moderate | moderate | moderate |
| White 2016 | moderate | Low | low | low | low | moderate | Moderate | moderate |

**Table S3:** Overview on the studies addressing whether intra- or postoperative measurements of PTH levels are superior for early detection of postsurgical hypoparathyroidism.

| **Statement:**  Best time-point of PTH measurements | **Number of studies** | **Number of patients included** | **References** |
| --- | --- | --- | --- |
| Postoperative measurements are superior | 8 | 652 | ^7, 26-32^ |
| No difference intraoperative *versus* postoperative | 3 | 392 | ^6, 33, 34^ |
| Intraoperative measurements are superior | 2 | 223 | ^35, 36^ |

**Table S4:** Overview of studies reporting various threshold levels for early postoperative detection of postoperative hypoparathyroidism. Apart from assay-related differences that were not addressed here this should demonstrate a large heterogeneity within the literature.

| **Threshold PTH level** | **Number of studies** | **Reference** |
| --- | --- | --- |
| 3 pg/ml | n=3 | ^62, 128, 148^ |
| 3.75 pg/ml | n=1 | ^191^ |
| 4 pg/ml | n=1 | ^156^ |
| 5 pg/ml | n=4 | ^24, 64, 71, 103^ |
| 6 pg/ml | n=1 | ^122^ |
| 7 pg/ml | n=2 | ^154, 156^ |
| 8 pg/ml | n=2 | ^39, 195^ |
| 8.02 pg/ml | n=1 | ^201^ |
| 9 pg/ml | n=3 | ^39, 156, 158^ |
| 9.4 pg/ml | n=3 | ^35, 126, 150^ |
| 9.5 pg/ml | n=1 | ^132^ |
| 9.6 pg/ml | n=1 | ^130^ |
| 10 pg/ml | n=17 | ^6, 23, 24, 32, 43, 47, 51, 62-71^ |
| 10,42 pg/ml | n=1 | ^120^ |
| 10.37 pg/ml | n=2 | ^44, 110^ |
| 10.6 pg/ml | n=2 | ^26, 41^ |
| 11 pg/ml | n=1 | ^134^ |
| 11.3 pg/ml | n=1 | ^101^ |
| 11.5 pg/ml | n=1 | ^21^ |
| 12 pg/ml | n=3 | ^34, 128, 178^ |
| 12.1 pg/ml | n=2 | ^146^ |
| 12.5 pg/ml | n=2 | ^115, 121^ |
| 13 pg/ml | n=2 | ^196, 202^ |
| 14.35 pg/ml | n=1 | ^105^ |
| 14.8 pg/ml | n=2 | ^41, 171^ |
| 14.82 pg/ml | n=1 | ^147^ |
| 15 pg/ml | n=11 | ^34, 44, 53-61^ |
| 15.39 pg/ml | n=1 | ^157^ |
| 16 pg/ml | n=3 | ^70, 133, 160^ |
| 16.8 pg/ml | n=1 | ^203^ |
| 18 pg/ml | n=2 | ^118, 155^ |
| 19 pg/ml | n=3 | ^125, 156, 165^ |
| 19.4 pg/ml | n=1 | ^161^ |
| 19.55 pg/ml | n=1 | ^105^ |
| 20 pg/ml | n=7 | ^46-52^ |
| 23 pg/ml | n=3 | ^32, 177, 192^ |
| 26.4 pg/ml | n=1 | ^176^ |
| 27 pg/ml | n=2 | ^95, 124^ |
| 30 pg/ml | n=3 | ^47-49^ |
| 39.8 pg/ml | n=1 | ^135^ |
| Values corresponded lower limit of the normal of PTH levels | n=8 | ^6, 47, 51, 53, 65, 67, 116, 128^ |
